# The NASSS framework for ex post theorisation of technology-supported change in healthcare: worked example of the TORPEDO programme

**DOI:** 10.1186/s12916-019-1463-x

**Published:** 2019-12-30

**Authors:** Seye Abimbola, Bindu Patel, David Peiris, Anushka Patel, Mark Harris, Tim Usherwood, Trisha Greenhalgh

**Affiliations:** 1 School of Public Health, University of Sydney, Sydney, NSW 2006 Australia; 2 Centre for Health Systems Science, The George Institute for Global Health, University of New South Wales, Level 5/1 King St, Newtown, NSW 2042 Australia; 3 Centre for Primary Health Care and Equity, University of New South Wales, Sydney, NSW 2052 Australia; 4 Westmead Clinical School, Faculty of Medicine and Health, The University of Sydney, Sydney, NSW 2006 Australia; 5 Nuffield Department of Primary Care Health Sciences, University of Oxford, Radcliffe Observatory Quarter, Woodstock Road, Oxford, OX2 6GG UK

**Keywords:** Ex post evaluation, Theory-driven evaluation, Diffusion of innovation, Scale-up, Programme sustainability, Implementation, Complexity of innovations, NASSS framework, Non-adoption, abandonment, scale-up, spread, sustainability framework, Innovation adoption

## Abstract

**Background:**

Evaluation of health technology programmes should be theoretically informed, interdisciplinary, and generate in-depth explanations. The NASSS (non-adoption, abandonment, scale-up, spread, sustainability) framework was developed to study unfolding technology programmes in real time—and in particular to identify and manage their emergent uncertainties and interdependencies. In this paper, we offer a worked example of how NASSS can also inform ex post (i.e. retrospective) evaluation.

**Methods:**

We studied the TORPEDO (Treatment of Cardiovascular Risk in Primary Care using Electronic Decision Support) research programme, a multi-faceted computerised quality improvement intervention for cardiovascular disease prevention in Australian general practice. The technology (*HealthTracker*) had shown promise in a cluster randomised controlled trial (RCT), but its uptake and sustainability in a real-world implementation phase was patchy. To explain this variation, we used NASSS to undertake secondary analysis of the multi-modal TORPEDO dataset (results and process evaluation of the RCT, survey responses, in-depth professional interviews, videotaped consultations) as well as a sample of new, in-depth narrative interviews with TORPEDO researchers.

**Results:**

Ex post analysis revealed multiple areas of complexity whose influence and interdependencies helped explain the wide variation in uptake and sustained use of the *HealthTracker* technology: the nature of cardiovascular risk in different populations, the material properties and functionality of the technology, how value (financial and non-financial) was distributed across stakeholders in the system, clinicians’ experiences and concerns, organisational preconditions and challenges, extra-organisational influences (e.g. policy incentives), and how interactions between all these influences unfolded over time.

**Conclusion:**

The NASSS framework can be applied retrospectively to generate a rich, contextualised narrative of technology-supported change efforts and the numerous interacting influences that help explain its successes, failures, and unexpected events. A NASSS-informed ex post analysis can supplement earlier, contemporaneous evaluations to uncover factors that were not apparent or predictable at the time but dynamic and emergent.

## Background

### The NASSS framework

The fortunes of technology programmes in healthcare are notoriously stormy [1–4]. Effective evaluation of such programmes requires in-depth, theoretically informed and mixed-method analysis that address potential challenges to adoption and scale-up [5–9]. Yet published evaluations are sometimes disappointingly theory-light, empirically superficial, and deterministic [5].

TG’s group undertook a narrative review of theory-informed frameworks for analysing and evaluating technology programmes in health and social care [10]. Previous research was synthesised and extended to develop a new evidence-based framework (abbreviated NASSS) for studying the adoption, non-adoption, and abandonment of technologies by individuals and the challenges to scale-up, spread, and sustainability of such technologies in healthcare organisations and systems [10].

**Fig. 1:**
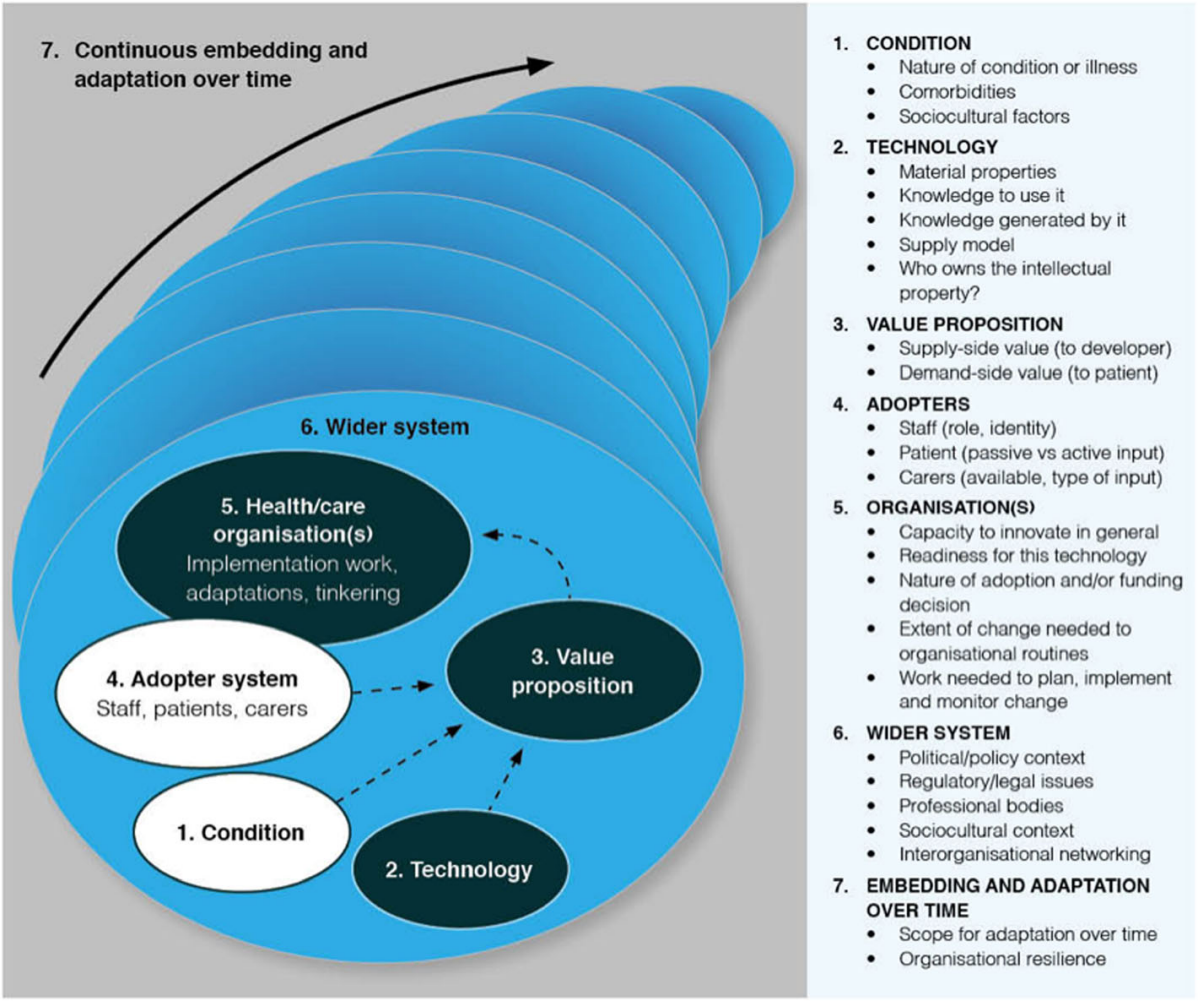
The NASSS framework for studying non-adoption and abandonment of technologies by individuals and the challenges to scale-up, spread, and sustainability of such technologies in health and care organisations (adapted from Greenhalgh et al. [10])

The NASSS framework is shown in Fig. 1. It consists of seven domains, each of which may be simple (few components, predictable), complicated (many components but still largely predictable), or complex (many components interacting in a dynamic and unpredictable way). The different sub-domains (right-hand panel in Fig. 1) can be applied eclectically to generate a nuanced narrative that surfaces different kinds of complexity in the unfolding programme.

Domain 1 is the illness or condition. Complexity occurs when the condition is metabolically volatile (e.g. diabetes in pregnancy), inherently unstable (e.g. drug dependency), poorly described or understood (e.g. a newly discovered syndrome), associated with co-morbidities (most commonly in elders), or strongly influenced by socio-cultural factors (e.g. poverty, social exclusion) [11].

Domain 2 is the technology. Complexity may relate to its material properties (e.g. functionality, dependability, speed), knowledge needed to use it, knowledge it brings into play (all technologies foreground some kinds of knowledge at the expense of others [12]), supply model (e.g. is it substitutable?), and intellectual property (how easy is it to say who “owns” this?) [13].

Domain 3 is the value proposition—both supply-side (value to the developer) and demand-side (value to the patient, healthcare system, and taxpayer or insurer). Complexity in this domain relates to difficulties in formulating a credible business plan (e.g. when efficacy or cost-effectiveness studies are unavailable or contested) [14, 15].

Domain 4 is the adopter system: the staff, patients, and carers who will be expected to use the technology (but who may refuse to use it or find they are unable to use it). Complexity may arise, for example, when the roles and practices assumed by the technology threaten professional traditions and codes of conduct [16].

Domain 5 is the organisation(s). Complexity in this domain may relate to the organisation’s general capacity to innovate (such things as leadership, resources, and clinician-managerial relationships) [17], its readiness for this particular technology (tension for change, balance of supporters and opponents) [17], the nature of the adoption and funding decision (more complex if it depends on inter-organisational agreements and speculative cross-system savings), potential disruption to existing routines [18], and extent of work needed to implement the changes (including gaining buy-in, delivering the change, and evaluating the change) [7].

Domain 6 is the wider system, including the policy context, support from regulatory or professional bodies, and public perceptions [5]. This domain also includes inter-organisational networking (for example, via quality improvement collaboratives), which can be a powerful way of spreading organisational-level innovations [17].

Domain 7 is embedding and adapting over time. Complexity may arise from the technology’s “brittleness” (inability to adapt to changing context) or from the organisation’s lack of resilience (inability to withstand shocks and setbacks through learning and adaptation) [19].

The NASSS framework is a sensitising device that incorporates and combines a range of existing theoretical perspectives on illness and disease, technology adoption, organisational change, and system change [10]. Empirical studies by TG’s team [20, 21] and others [22, 23] have demonstrated how NASSS can help construct a rich narrative of an unfolding technology programme and identify the various uncertainties and interdependencies that need to be contained and managed if the programme is to succeed. To date, the NASSS framework has not been used retrospectively for secondary analysis of a historical dataset. In the remainder of this paper, we first offer a theoretical justification for using NASSS for ex post analysis of case study data. We then describe the TORPEDO research programme (an initiative to introduce a new technology, *HealthTracker*, into Australian primary care) and the large empirical dataset it generated. We describe how we applied the NASSS framework to the TORPEDO secondary dataset and to a new sample of primary interviews with programme staff. In the “Results” section, we present our ex post analysis and highlight how this approach allowed us to draw together and extend previous analyses of different parts of the dataset. In the “Discussion” section, we offer reflections (theoretical and methodological) on our findings.

### Ex post theorisation of complex case studies in healthcare

In 2011, in the introduction to “Explaining Michigan” (a retrospective analysis of the somewhat unexpected success of a state-wide quality improvement programme), Dixon-Woods et al. observed: “Understanding how and why programmes work—not simply whether they work—is crucial. Good theory is indispensable to advancing the science of improvement.” (page 167) [24].

The Michigan Keystone Project achieved a dramatic reduction in central venous catheter bloodstream infections in more than 100 participating intensive care units across the state [25]. It was widely hailed as a model of good practice (for example, by the World Health Organization, who sought to roll it out internationally) [25]. But initial accounts of the programme were simplistic and superficial, and helped perpetuate the myth that success was attributable to a “simple checklist” rather than to a highly complex and context-specific social intervention [24].

Dixon-Woods et al. argued that because programmes almost never proceed as planned, we rarely discover why a programme succeeded (or failed) by studying the initial study protocol (which can tell us only how its architects *assumed* it would work) [24]. The “why” question must be addressed through a combination of re-analysis of primary data and additional interviews with programme staff and evaluators, who are asked to reflect *retrospectively* on what happened [24]. Such an analysis tracked the Keystone Programme’s success to a combination of six powerful and synergistic social influences: isomorphic pressures to join the programme and conform to its requirements, a professional community of practice maintained via weekly teleconferences, attention to the social and behavioural aspects of the intended change, nurturing a culture of commitment to quality improvement, harnessing data on infection rates as a disciplinary force, and judicious use of hard-edged formal accountability. This rich and innovative theorisation was later applied prospectively to explain why the same checklist-based intervention was much less successful in achieving its quality improvement goals in a UK setting [26].

Dixon-Woods et al.’s work challenged a tradition in which quality improvement programmes were typically presented in the academic literature as sanitised, logic-model accounts of how programme goals were met through rigorous adherence to predefined protocols. In the real world, goals are met because (and to the extent that) humans solve problems creatively and continually adapt the official blueprint to match what is acceptable, feasible, and affordable locally. The messiness of implementation, and the all-important question of *how* local actors became inspired, carved out the necessary time and managed local contingencies, and stakeholder politics are usually stripped away in the final report in the misguided pursuit of spurious scientific ideals (parsimony, objectivity, generalisability). Yet it is these messy, local narratives that could reveal *why* the programme worked in some settings some of the time but not in other settings or at other times.

## Methods

### Overview

**Fig. 2:**
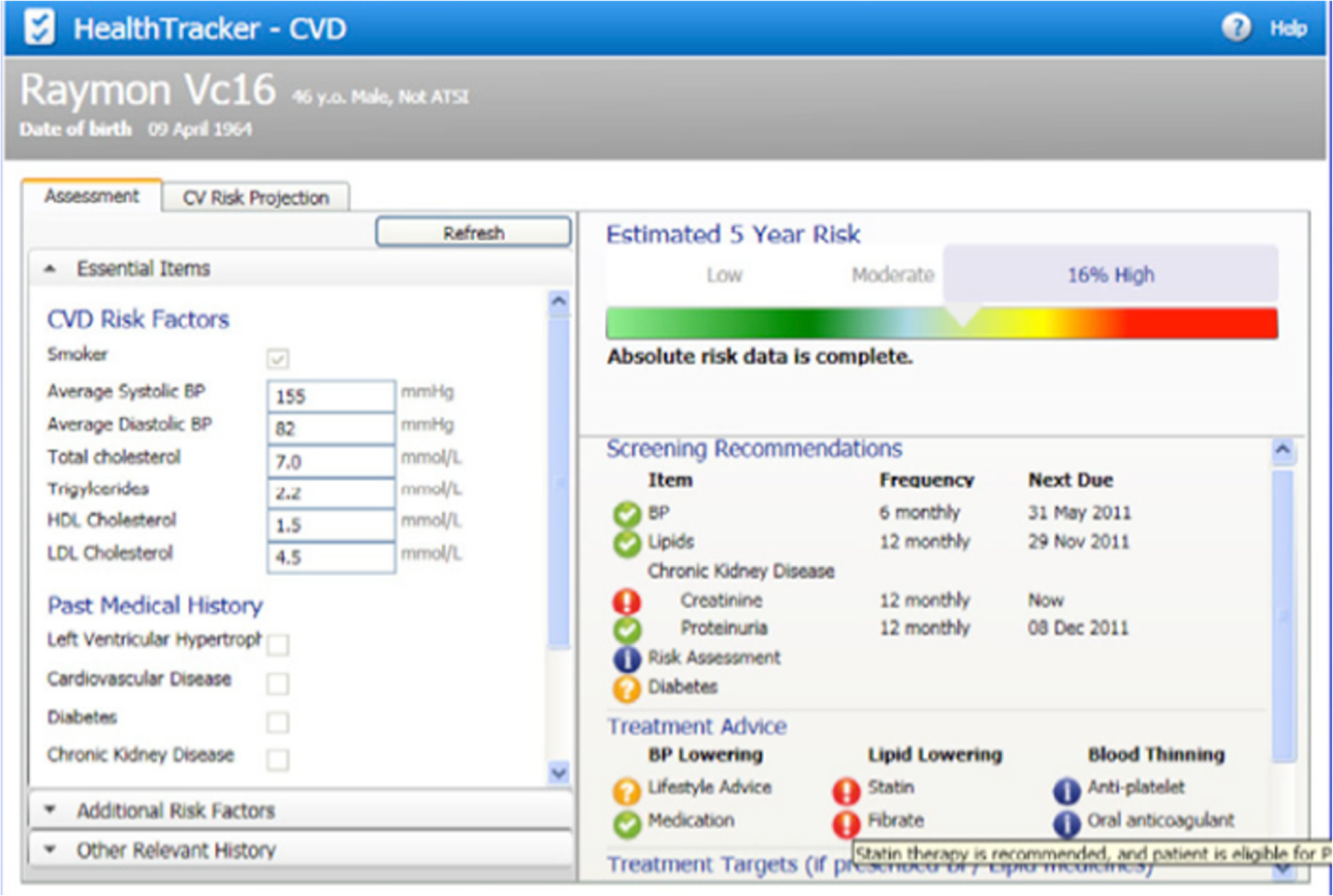
Screenshot of the *HealthTracker* technology

We studied the TORPEDO (Treatment of Cardiovascular Risk in Primary Care using Electronic Decision Support) research programme, a multi-faceted computerised quality improvement intervention for cardiovascular disease prevention in Australian general practice, using a technology by the name *HealthTracker* (Fig. 2). This study was conducted using primary (i.e. ex post interviews of TORPEDO researchers) and secondary (i.e. datasets and publications to date from the TORPEDO project) datasets. Based in Australia, TORPEDO extends back more than a decade. Against a context of high rates of cardiovascular disease in certain groups (notably, the indigenous Aboriginal population), researchers at the George Institute for Global Health in Sydney began an initiative to improve opportunistic risk factor assessment and guideline-recommended primary and secondary prevention for individuals at high risk of cardiovascular disease.

### The *HealthTracker* technology

*HealthTracker* is a third-party add-on software tool which incorporates ten different clinical practice guidelines into a single on-screen algorithm. It sits on the clinician’s desktop and draws data from the patient’s electronic medical record to populate a quantitative risk estimate (represented visually) for future cardiovascular disease. In patients who are at high risk of cardiovascular disease, it may recommend further testing, medication (statins, antihypertensive drugs, and antiplatelets), and lifestyle changes. The point-of-care decision support is aided by a “traffic light” (red, amber, and green) visual alert to flag missing information or suboptimal treatment and prompt conversations with patients. An audit tool (designed for use by both clinical and non-clinical managers) allows aggregation of performance data across the practice and access to a web portal for comparison with other de-identified sites.

The idea for the study emerged from a workshop on the NASSS framework at the George Institute in Sydney in 2018, in which TG invited participants to share examples of health technology projects that had met with implementation challenges. AP (a cardiologist and chief investigator of the TORPEDO programme), DP (a GP and research lead on TORPEDO), SA (a health systems researcher), and BP (at the time, a completing PhD student who had been project manager on TORPEDO) all contributed to that workshop.

It was evident that the application of NASSS to the extensive TORPEDO dataset had the potential to synthesise and extend insights from previous evaluations about whether and to what extent *HealthTracker* had succeeded. Accordingly, we agreed to revisit the original dataset and undertake some additional interviews to capture retrospective reflections of the study team. The principles and methodology of Dixon-Woods et al.’s ex post theorisation required little adaptation when we sought to apply them to the *HealthTracker* programme.

### Primary dataset: ex post interviews

SA and TG (who were not involved in the original empirical work) read the published papers on TORPEDO and drafted an initial set of questions based on the NASSS domains. They then consulted BP, who had been involved in TORPEDO from the outset and whose doctoral work had been an evaluation of the TORPEDO programme. With her input, SA and TG refined the draft questions to construct the specific “ex post” questions below:

- What was the nature of the conditions for which *HealthTracker* was developed? What disease-related uncertainties, co-morbidities, and cultural influences did the programme grapple with?
- How did *HealthTracker*’s material features (e.g. functionality, dependability, interoperability, and customisability) and its supply model influence its uptake and use in different settings?
- What value did *HealthTracker* generate (for developers, patients, providers, and the healthcare system more widely)? Were there weak links in the value chain—and if so, why?
- What explained situations in which an individual resisted adopting *HealthTracker* or abandoned it after a short trial period?
- To what extent could variation in adoption and mainstreaming of *HealthTracker* be explained by organisational antecedents, organisational readiness, nature of funding decision, degree of disruption to organisational routines, or the organisational work needed to normalise and evaluate the technology?
- To what extent could variation in uptake and use of *HealthTracker* be explained by an adverse (or supportive) external context—in particular in the policy, regulatory, professional, or public realm? How strong were inter-organisational networks and to what extent did they enable participating organisations to deliver change?
- What changed over time (in individuals, in organisations, in the wider system)? To what extent could the technology, the service model, and the organisation adapt—and why?

We collected a primary dataset of five interviews with the programme evaluation team, comprising the principal investigator of TORPEDO (AP), three chief investigators (DP, TU, and MH; the last two were GPs with an interest in decision-support software development), and one project manager and PhD student (BP). The ex post research questions listed above were used as prompts in a conversational interview in which the participant was invited to tell the story of the TORPEDO programme in their own words and reflect on the multiple interacting influences on it. Interviews were audiotaped with consent and professionally transcribed.

### Secondary dataset: the TORPEDO research programme

Publications from TORPEDO are summarised in Table 1, which shows their empirical focus, dataset, and theoretical contribution. Each was produced for a different audience and had a different focus; together, they provide a rich and multi-faceted picture of the programme’s origins, rationale, and unfolding fortunes over a 10-year period. Along with relevant primary source material from those original studies (in particular, a re-analysis of 19 transcripts of interviews with health professionals and managers using the NASSS framework), these constituted the secondary dataset for our ex post analysis. In the following paragraphs, we provide brief descriptions of the publications reporting the original studies.

**Table 1:** Summary of publications from the TORPEDO programme

Paper	Empirical focus	Subset of data analysed in this paper	Theoretical contribution
Peiris et al. [27]	Development and validation of *HealthTracker* software for risk factor measurement and management	Development sample: 137 patients in 1 practice. Validation sample: 21 GPs from 8 practices and 3 Aboriginal Medical Services generated data for 200 patients	Clinical validity and reliability of the technology. Comparison with existing gold standard statistical algorithm
Peiris et al. [28]	GPs’ experience of using the *HealthTracker* technology in a clinical setting	21 qualitative interviews with participating GPs	Technology-in-practice lens. Knowledge from the tool was combined pragmatically in real time with intuitive and informal knowledge from GPs’ professional networks and wider clinical and patient priorities
Patel et al. [29]	Protocol for mixed-methods process evaluation for RCT	N/A	Multiple evaluation theories considered: Logic model using RE-AIM (reach, effectiveness, adoption, implementation, maintenance) Realist evaluation Normalisation process theory Theoretical domains framework
Peiris et al. [30]	Cluster RCT of *HealthTracker* vs usual care in Australian primary care	60 sites randomised (30 in each arm). Descriptive data on uptake and use of the technology and patient process/outcome measures	Effect size. Compared to control arm: 10% increase in percentage of eligible patients receiving appropriate and timely measurement of cardiovascular risk factors (statistically significant) Small increase in percentage of people at high risk of cardiovascular disease receiving recommended medication prescriptions (not statistically significant)
O’Grady et al. [31]	In-depth qualitative study of risk communication	Video ethnography of a single case, analysed using multi-modal linguistic ethnography	Interactional socio-linguistics: the computer as a social and material “actor” in a complex communicative encounter
Patel et al. [32]	Post-trial real-world implementation study	41 sites included (from 60 of the original sample). Quantitative process and outcome measures as for RCT	Sustained overall effect: evidence of continued risk factor testing and improvements in prescription of evidence-based preventive medication with significant benefit for the undertreated high risk patients
Patel et al. [33]	Mixed-methods process evaluation of the RCT	Purposive (maximum variety) sample of 6 sites agreed to participate in the process evaluation. Quantitative process measures included attitude to technology survey (*n* = 32 GPs from 21/30 intervention sites). Qualitative process measures included 19 health professional interviews.	Variation in use of *HealthTracker* or patient outcomes was not explained by team climate or job satisfaction. Normalisation process theory informed a thematic analysis which identified 4 influences on technology uptake: organisational mission, leadership, collaboration, and unintended material consequences of the technology

The development, core functions, and early usage statistics of *HealthTracker* were described in a technology journal in 2009 [27]. A qualitative pilot study of this tool, based on ethnography and interviews and analysed using a technology-in-practice lens, was published in a sociological journal in 2011 [28]. This study highlighted the gap between the abstract evidence inscribed in the technology and the realities of real clinical cases (which were often unique, messy, and complex).

Based on this pilot work, a protocol was developed for a randomised controlled trial (RCT) of *HealthTracker* with mixed-method process evaluation [29]. The trial (Australian Clinical Trials Registry 12611000478910), which randomised 60 sites (general practices or Aboriginal Community-Controlled Health Services) to intervention (installation of *HealthTracker* and support to use it) or usual care, was reported in 2015 [30]. In practices randomised to the intervention, patients were more likely to have risk factors measured and receive evidence-based preventive care than those in control practices. Absolute differences between intervention and control arms were small, but differences in the primary endpoint for appropriate screening of cardiovascular risk factors were statistically significant. High-risk patients in the intervention group whose care had been suboptimal prior to the study showed clinical benefit.

Some risk assessment consultations using *HealthTracker* were video-recorded and analysed using multi-modal conversation analysis, producing, for example, a book chapter on the nuanced challenges of using technology to help communicate risk in patients with variable health literacy and (sometimes) a very different world view from the doctor [31].

A post-trial follow-up study of 41 of the original 60 sites (from both arms of the trial) assessed the use of the *HealthTracker* technology under real-world conditions. The initial paper from that follow-up study reported mainly quantitative data (e.g. proportion of doctors using the tool; proportion of patients being treated according to guidelines) [32]. But this on-average finding obscured the fact that in the implementation phase, use of *HealthTracker* to support proactive preventive care varied widely between individual clinicians and practices. A mixed-method study, based on surveys and semi-structured interviews, explored reasons for this variation [33]. This paper concluded that four spheres of influence (discussed further below) appeared to account for the fortunes of the *HealthTracker* technology in a practice: organisational mission and history, leadership, team environment, and material properties of the technology.

### Ex post analysis

Using the NASSS domains as a sensitising framework, we undertook a thematic analysis of the previous TORPEDO publications along with the new primary interviews. As we amassed material under each domain, we drew on various theoretical lenses (some identified in a previous systematic review of technology evaluation frameworks [10] and others known to the research team), which are highlighted in the next section where we present our analysis, along with our key findings, including particular theories mobilised to explain specific findings. Each of the questions and discussion points during the ex post interviews requires the application of theory, and different theories were appropriate for exploring the domain of the NASSS framework.

The re-analysis of primary interviews included a search for disconfirming data (i.e. searching the dataset material, both quantitative and qualitative, that might challenge our emerging interpretation of what happened) to ensure that we were not simply cherry-picking quotes that supported our interpretation. We included ten quotes from the original dataset, all selected by BP, and checked by SA and TG using the principle of “apt illustration”. Whilst we did not undertake further quantitative analysis for this study, the original quantitative findings from TORPEDO—and in particular, differences that were and were not statistically significant in the randomised trial [30]—were a major focus of discussion in the ex post interviews.

A provisional summary of findings, produced by SA, BP, and TG, was discussed and factual errors corrected. Differences of interpretation were resolved by discussions among the research team and by triangulating the findings with historical qualitative and quantitative data from the primary TORPEDO studies. For example, when one of the ex post interviewees mentioned that negative media reports about statins may explain a drop in prescriptions during the follow-up study, we checked if this explanation was supported by the quantitative trend data from the follow-up study and from GP interview transcripts from the original studies, both suggesting that GPs were influenced by the media reports (see “The intended adopters” under the “Results” section).

## Results

The analysis identified interacting complexities in the TORPEDO programme which played out differently in different sites and settings. These are presented under the NASSS domains below. Direct quotes from the new primary dataset of ex post interviews are labelled “ex post interview [number]”; quotes from the original TORPEDO dataset are labelled with the original coding notation (e.g. 2282-005, with the first four digits indicating the original study site number and the last three digits indicating the participant number).

### The condition

*HealthTracker* was designed for use in two kinds of patient: those who already had cardiovascular disease and those (usually asymptomatic) who were potentially at high risk of developing it. Established cardiovascular disease is well characterised, and guidelines for its management are relatively uncontested and widely accepted. The evidence base on managing cardiovascular risk is more complex. It is skewed towards a white European and North American population (especially the US Framingham study, on which the *HealthTracker* algorithm was partially based). Furthermore, since cardiovascular risk is a continuous variable influenced by multiple risk factors such as blood pressure and cholesterol levels, *HealthTracker* could not offer an unambiguous binary categorisation of patients into “high risk” or “low risk”.

In Australia (as elsewhere), cardiovascular disease is strongly patterned by socio-demographic factors: it is commoner in those who are poor, those with low health literacy, and in Aboriginal people. Such individuals are more likely to have comorbidities such as diabetes or mental health conditions (“High risk is a sort of multifarious set of component conditions”—ex post interview 2). They may also have cultural beliefs and practices that affect their ability and willingness to understand the risk communication and comply with preventive treatment.

The risk communication tool in *HealthTracker* worked well for many patients (“I found the patient education information just great, it was just wonderful.”—Nurse in TORPEDO study, 2368-005). But it assumed that patients would be able to understand a visual representation of quantitative risk and make a rational decision to alter their lifestyle based on it. This was not always the case, partly because of numeracy (“I find that they’re [absolute risk percentage] harder to explain to the patient with a number. So we need to go back and look at how to translate into the number needed to treat. But that’s very hard concept for the patient to understand at the moment too.”—GP in TORPEDO study, 2290-001), and partly because of competing priorities in complex lives (“[*HealthTracker*] works for people that have structured lives … but some patients are much less organised, and they have social and other medical problems … which interfere with their ability … to accept and to seek out systematic care”—ex post interview 4).

The TORPEDO findings confirmed that cultural habits die hard and familiar folk models of illness and risk may over-ride less familiar epidemiological ones [34]. A 66-year-old patient with a family history of cardiovascular disease and adverse clinical and lifestyle factors commented:

... it’s no good saying we’ll change your lifestyle. I’m 66 years old. I have a lifestyle, you know. I’m not an alcoholic. I don’t over-drink. I don’t you know I don’t overeat. I’m just, just a big bloke. Look, I didn’t walk out of there thinking, oh, I’m only going to eat salad and, you know, drink water for the rest of my life.—Interview with patient in TORPEDO study [31]

The “no symptoms, no problem” mindset helps explain why patients with established cardiovascular disease appeared to engage better than those flagged for primary prevention (“I have this difficulty convincing patients that they should be on medication when the indication is only based on high risk … ‘Doctor, but I haven’t got the problem now so why do you want to give me the medication’ and, of course, medications are not cheap”—GP in TORPEDO study, 2308-001).

### The technology

*HealthTracker* had many attractive design features (“[GPs] loved it, … loved the traffic light [which] was simple, [and] loved seeing the graphs, looking at the heart age over time”—ex post interview 1). Many valued the way it structured care (“*HealthTracker* reminds me what needs to be followed, to be checked and followed. So it wasn’t so much telling me what the guidelines are, it was telling me what I needed to do to ensure that their health, everything’s been covered”—GP in TORPEDO study, 2303-001). But GPs described technical glitches (such as when data on the patient’s record did not appear in the HealthTracker viewer) that were frustrating and interfered with their use of the technology in real time. Many found themselves regularly on the phone to the helpdesk. A major concern was that “apps that would just chew up memory, make the EMR [electronic medical record] run slowly; people said, ‘I don’t want to have anything to do with this thing, because it’s making my existing work flow worse’” (ex post interview 2).

Despite its visual appeal, “the user design wasn’t very good” (ex post interview 3), and in retrospect, findings suggest that it was not fit for purpose. It was not easy to integrate *HealthTracker* into existing workflows and practices for quantifying risk, advising patients, and prescribing medication. *HealthTracker* appeared in a side bar on the GP’s screen with pop-up prompts, “and sometimes prompts would go up, [or] wouldn’t; some of them wouldn’t see it because they would [mistakenly] shut it off, [and] … would say, oh, it’s gone, I don’t know where it is.” (ex post interview 1). Because of these technical imperfections, GPs participating in the TORPEDO study soon divided into highly motivated and/or technically adept ones, who persisted with the technology, and the rest, who gave up on it (“We tried to fix it and it didn’t work, then I just stopped doing it, yeah. We never knew why, I don’t know if it is the software because we tried many times … It’s never worked.”—part-time GP in TORPEDO study, 2290-003).

*HealthTracker*’s inbuilt algorithms foregrounded “hard” risk data (biometrics, family history) at the expense of “softer” data (e.g. on personal and cultural context) that could have informed a more individualised approach to care [27]. This was a conscious design feature, but it helps explain why, glitches aside, different GPs had very different levels of use of the tool (see domain 4).

The TORPEDO project team sought to facilitate adoption by ensuring from the outset that *HealthTracker* was able to integrate with more than one electronic record system. Whilst it covered only two such systems, they amounted to 80% of the Australian market. But the integration was only one way: “nothing from *HealthTracker* populated into the EMR; [only] the reverse occurred” (ex post interview 3). This meant that the risk score and management plan did not automatically populate the patient’s record—a feature that contributed to clinicians’ experience of “clunkiness”.

### The value proposition

*HealthTracker* was developed in a university setting by publicly funded research. There were two implicit potential models for introducing it into Australian primary care. The technology could be sold directly to GP practices or paid for by or through government entities, e.g. Primary Health Networks (PHNs), which are the organisations responsible for planning and commissioning primary care services, one for each of 31 geographically defined locations across Australia; and Medicare, which is the publicly funded universal healthcare system in Australia. The value proposition varied accordingly. The Australian government is prioritising digital health initiatives (see “The wider system” section). To government as a third-party payer, the potential value of *HealthTracker* would be “quality of care, [a] better performing health system, reduced inefficiency, better use of medicines, … reduction of morbidity and mortality, and no unintended safety consequences” (ex post interview 2). In addition, there was hope among the TORPEDO team that *HealthTracker* would support a shift towards a more prevention-oriented healthcare system. One researcher commented that the Australian primary healthcare system is designed to be “… reactive, not proactive, and what we’re trying to do [by introducing HealthTracker] is to graft on some extra things that make it more proactive” (ex post interview 4).

The TORPEDO team also anticipated that the value to Primary Health Networks (which at the time were known as Medicare Locals) would be in the form of improved workflow and easier audit and performance management within GP practices. A modelled cost effectiveness analysis showed a small but statistically significant reduction in clinical risk factors within a PHN population based on the TORPEDO trial data, suggesting a small economic benefit from preventing CVD events (paper submitted). The economic evaluation showed that if *HealthTracker* were to be scaled up to a larger population, the intervention has potential to prevent major CVD events at under AU$50,000 per event averted. However, at the PHN level, investment decisions for commissioning similar interventions based on cost-effectiveness analyses are scant.

The heavy burden of preventable and costly cardiovascular disease, particularly in Aboriginal communities, made the value proposition particularly compelling for community leaders. One researcher recalled, when recruiting the Aboriginal Community-Controlled Health Services to the TORPEDO trial, “it was just so pressing how – every one of the [community] board members, either themselves or relatives, knows someone who’s died of heart disease, or stroke, or diabetes, or kidney diseases; it’s just absolutely everywhere” (ex post interview 2).

Some individual GPs shared this perspective, viewing *HealthTracker* in positive value terms as supporting better (more proactive) care and making it easier and quicker to follow evidence-based guidelines and monitor their own performance. They felt it could potentially save them time “because it got all sorts of information out of the medical record and told you what otherwise you have to go hunting for” (ex post interview 5). Because *HealthTracker* synthesised several guidelines so as to streamline decision-making in patients with multi-morbidity, it saved considerable time sourcing individual guidelines.

But this would generate value only for GPs who were committed for professional reasons to delivering guideline-informed care, since *HealthTracker* increased overall consultation length [33]. The conversation triggered by the risk visualisation tool could sometimes be lengthy (“the thing is, it’s not time to run the programme, it’s time to actually chat to the patient. So if you’re going to go through all this, you’ve got to be prepared to have a good 10 minute chat with the patient because you actually want to engage them and help them understand where they’re at and make a difference and that’s the time.”—GP in TORPEDO study, 2282-005). Since the Australian payment system predominantly rewards GPs on a fee-for-service basis rather than (say) incorporating a pay-for-performance scheme (as in the UK Quality and Outcomes Framework [35]), the technology could be viewed as having negative financial value for GPs, especially given the many technical “bugs”, which could be time-consuming to resolve.

The value of *HealthTracker* to patients was complex and varied for different individuals and communities. For example, only a minority of patients valued the focus on prevention and future health gain: “patients who are not at high risk, who are motivated and got high health literacy, are the minority, [while] the majority of patients at high risk have got multiple problems and need much more hands-on working” (ex post interview 4). And some GPs had commented that using *HealthTracker* would increase their professional status in the eyes of current or potential patients—“that you’re a 21st century doctor and you’re doing the right thing” (ex post interview 4).

However, patients’ main priority when choosing a GP was not always quality of care delivered. For example, in ethnically diverse areas of Sydney, “[a large proportion] of the GPs consult in a language other than English, people find the same language, same culture GPs, … that’s what people are looking for, they’re not necessarily looking for them following guidelines” (ex post interview 4). Some patients were driven predominantly by material needs. The Australian copayment system meant that out-of-pocket payments for a GP consultation could be $30–$50, which might place negative financial value on additional medication and GP appointments triggered by a *HealthTracker* focused consultation.

### The intended adopters

In the TORPEDO study, at least 1 GP responded to a survey in 21 of the 30 intervention sites; of these, fewer than one third said they used *HealthTracker* for more than half of eligible patients, even though most expressed positive attitudes to the technology (e.g. they considered it easy to use, valued the data it generated, and felt it helped improve the quality of care) [32].

Their reasons for limited adoption were complex; they included technical issues described in domain 2. Those aside, *HealthTracker*’s potential to prompt the screening of asymptomatic patients for cardiovascular disease risk was “a low hanging fruit that most GPs were happy to engage with and could see that was an important thing to do” (ex post interview 2). This partly explains why the intervention arm of the TORPEDO RCT showed significant improvement in process measures (measuring and documenting risk factors). But despite this, there was no significant improvement in prescribing preventive drugs in the TORPEDO trial. At the level of individual consultations, GPs may have been taking account of the (often complex) socio-cultural factors described in domain 1 when judging whether to use *HealthTracker* at all and (if they did) whether to follow the algorithm’s recommendations.

Limited adoption of *HealthTracker* was also, TORPEDO researchers hypothesised, because there was a mismatch between the recently published recommendations for primary prevention of cardiovascular disease inscribed in the software (based on formal guidelines) and more intuitive prevailing assumptions about what was good practice (based on collectively shared practical wisdom known as *mindlines* [36]). For example, GPs may have withheld medications because of anticipation of poor adherence or history of non-adherence. Also, negative media reports about statins at the time of the study [37] may have made some GPs more cautious, especially when managing patients who were high risk but without established disease. TORPEDO data showed that whilst statin prescription increased among those with a diagnosis of cardiovascular disease, it fell for those without such a diagnosis: “I think there was a huge drop in the prescription for statin … Lipitor came into the news around that time. … And I quickly had a look and realised that yes, I did reduce the prescription of the statin … [and] the prescription for high blood pressure group may have dropped at the same time, not just the statin.” (GP in TORPEDO study, 2290-001).

*HealthTracker* also appeared to exert what one researcher called “psychic costs” in the form of anxiety induced by a red light which alerted GPs to recommendations they did not follow [38]. GPs felt they were being marked down and expressed along the following lines: “don’t tell me to do something when I’ve made an active decision in discussion with my patient to not do it, don’t keep giving me a red traffic light” (ex post interview 3). In contrast, a GP researcher who was part of the TORPEDO team said: “I found it very useful, every time I saw a patient, I’d open the *HealthTracker* and have a quick squizz, and make sure that there were no red indicators anywhere” (ex post interview 5).

### The organisation(s)

Not all practices invited to participate in the TORPEDO study chose to do so. And among the studies that participated in the trial, 15 declined to participate in the post-trial study because of the following: the service was closing or moving (4 practices), concerns that *HealthTracker* would slow down their computer system (3), limited resources (3), changing to an incompatible electronic record system (3), already using another cardiovascular risk tool (1), and lack of interest (1) [32]. In other words, the practices which declined to participate may have had significant organisational-level issues to report, and findings from the practices which *did* participate may not reflect all those issues.

There was wide variation in participating practices’ underlying capacity to innovate. Technical infrastructure was sometimes poor, increasing the likelihood of technical crashes (“some practices don’t tend to change their hardware very often, or let it upgrade very often, so you’re trying to run sophisticated new software on older machines”—ex post interview 5). Some larger GP practices and Aboriginal Community-Controlled Health Services (ACCHSs) had “been engaged in quality improvement work very strategically for about 15 years [and] already had an operational structure that they could weave [*HealthTracker*] into” (ex post interview 2). In some, there was a dedicated individual focused on audit and quality improvement (“we can report to them that, you know, for example only 30% of the high-risk patients are being prescribed with triple therapy and they go, whoa.”*—*Health information officer (ACCHS) in TORPEDO study, 2282-001). Notably, some of the more confident larger practices sought a high degree of autonomy over how and when *HealthTracker* was used.

Larger practices sometimes also had an on-site IT support person or technically adept practice manager who could troubleshoot problems and coordinate remotely with the developers. At the other end of the spectrum were small, poorly resourced practices, who “had less experience doing this sort of thing, [and] probably needed a bit of arm twisting to sign up” (ex post interview 2). In extreme cases, the practice was not even able to install the software. More commonly, a “series of cascading negative things [could] then lead to complete abandonment”.

Whilst practice size was to some extent a proxy for capacity to innovate, the latter was also influenced by the practice’s governance structure [39, 40]. In small one- or two-doctor practices, decision-making was generally very streamlined. In a typical GP practice, quality improvement is commissioned by Primary Health Networks (PHNs) and practices are facilitated to conduct audits of their electronic medical records and provide de-identified data to the PHN. Each PHN is governed by a board, but there are hundreds of GP practices within a PHN region. Thus, the owner of a small practice was a GP who was essentially the CEO and the provider, such that “once you’ve engaged the principal or principals, and if they’re taken with the idea, then they’ll just do it” (ex post interview 4). This also explained why small practices could sometimes (albeit relatively rarely) overcome capacity disadvantages (“I’m probably taking about 90% of the data cleaning here, in this surgery.”—GP in TORPEDO study, 2290-001).

In larger organisations, several levels of governance were involved. In ACCHSs, for example, there were three tiers of decision-makers: “[The first tier is] community elected broad members, … the next tier is about senior management support for it, that’s the CEO and their senior level staff, and then the next tier is the providers or clinicians. …. We wouldn’t be able to work with any service without having all three of those processes in place” (ex post interview 2). Whilst strategic-level actors tended to make decisions on the basis of population disease burden and likely long-term benefit, operational-level actors appeared to be more concerned about short-term costs and workload implications and the factors discussed in domain 4.

Larger GP practices required greater coordination and aligned governance structures to facilitate the organisational change that was necessary for adoption, and this depended on competing priorities and staff continuity (especially in training practices with a high turnover of registrars). There was sometimes a mismatch of priorities between the “entrepreneur” GP (or, occasionally, a practice manager), who made the decision to sign up for the trial and embraced the technology with enthusiasm, and other staff (fellow GPs and most practice managers) whose engagement was often much lower. As the TORPEDO researchers discovered, “when you sign on a [large] GP practice … usually agreed by the lead GP who may be enthusiastic about intervention, … it really needs all the GPs to be committed and want to use it” (ex post interview 3).

Larger practices had a more diverse and distributed workforce. Potentially, this could reduce the cost of adoption of *HealthTracker*, for example, if nurses rather than doctors undertook the risk assessment (as happens routinely in the UK [41]). But large practices typically have a clear division of labour (with formal job descriptions, for example), so optimal embedding of new technologies may require revision of roles and routines and regular retraining. In some cases, *HealthTracker* work could not be sustained if a key member of administrative staff was absent. Given the high staff turnover in larger practices, community health workers (e.g. Aboriginal health workers, who already undertook some screening and health education tasks) could potentially “spend more time explaining to [patients] what it [the HealthTracker data] was all about, talking to them about lifestyle changes, their medication, why they need to be on them, how they could continue taking them and supporting them to do that” (ex post interview 4). Unfortunately, use of *HealthTracker* could not be easily incorporated into community health workers’ role in some ACCHSs for several reasons including lack of access privileges, low health worker confidence in use of computers, perceived time constraints, low GP confidence in health workers, and governance issues (“they weren’t given the green light by the head of the board”—ex post interview 1).

Variation in capacity to innovate (a phenomenon we have documented previously in GP practices involved in complex intervention trials [42]) raised the question of whether and how much to support each GP practice to implement *HealthTracker* during the TORPEDO trial and subsequent real-world implementation. This was partly for cost reasons (“it would have taken an extra couple of years [of planning] and another million dollars or something; it’s not cheap to do this kind of stuff”—ex post interview 4) and partly because of concerns that too much external support would limit the external validity of the findings. For these reasons, TORPEDO researchers decided to implement the intervention in a more or less standardised way.

Some of these findings, based on the NASSS framework, are resonant with those of an earlier theorisation using normalisation process theory, which identified four key influences on the routinisation of *HealthTracker* in participating practices: organisational mission and history (e.g. strategic investment to promote a culture of quality improvement), organisational leadership (e.g. ability to energise staff), team environment (e.g. extent to which team members with different skill sets worked in complementary ways), and technical features of the tool (covered in domain 2) [33].

### The wider system

*HealthTracker* was not classed as a medical device so did not require regulatory approval. Technology vendors saw regulation as a two-edged sword. On the one hand, lack of regulatory hurdles meant that it was easier to get them to market. On the other hand, achieving regulatory approval, had it been required, would have given the vendor an advantage over competitors.

The TORPEDO team was keen to create an institutional environment that would promote the use of *HealthTracker* by GP practices. They sought to position *HealthTracker* nationally so that it could generate revenue for GPs and GP practices in the future.

For example, they sought to maximise the chance that professional bodies supported and endorsed its use: “We made a decision very early on that that we would just use [existing] guidelines, whether or not we agreed with the guidelines” (ex post interview 3). This strategy was based on the assumption that if the guidelines emanated from professional societies, most physicians would accept them as reasonable. They had anticipated a potential scale-up platform through the Royal Australian College of General Practitioners (RACGP) and had selected the technology developer because of its existing relationship with RACGP (“we were somewhat lured into the attraction of working with them [the developers], because they’d signed this partnership with the College of GPs … to make this software available to all 20,000 members of the College of GPs”—ex post interview 2). However, RACGP subsequently discontinued this partnership because of negative feedback from its members, especially in relation to the tool slowing down practice systems. Even though RACGP had a long history of endorsing clinical practice guidelines, they did not endorse *HealthTracker* to their members. This was partly because “when it comes to endorsing software, that’s a relatively new space for them; [they] approached it like a guideline, … and missed the point that we weren’t trying to create a new guideline; we were trying to implement existing guidelines” (ex post interview 2).

By targeting an institutional level higher than professional organisations (i.e. government), the TORPEDO team sought to alter the rules that govern recognition and reimbursement of the use of software in delivering health services more broadly. The team had initially sought to list the use of *HealthTracker* on the Medicare Benefits Schedule (MBS), the government-subsidised health services, given Australia’s fee-for-service remuneration model for GPs. But this approach stalled initially: “we put in a submission to the federal government only to be told eventually that from a legislative viewpoint, MBS items can’t be attached to software” (ex post interview 3).

PHNs have the mandate to facilitate quality improvement programmes as part of their work, with dedicated staff to support that work, though such programmes do not tend to be focused on particular technologies. The TORPEDO researchers hoped to use the results of the trial “to drive the decision-making process a little bit more rationally” (ex post interview 2). This was particularly important at the time, given the absence in Australia of other quality incentives to promote proactive care for people at risk of cardiovascular disease. Without such incentive programmes, or the ability to bill patients or insurers for using *HealthTracker* and similar software, the chances of widespread adoption and scale-up of *HealthTracker* are probably limited.

The TORPEDO researchers built inter-organisational communication and networking into the study design. It is well established that complex innovation in healthcare is facilitated when different organisations communicate with one another, share experiences, and resources, and progress a shared vision of what they are collectively trying to achieve—perhaps using the quality improvement collaborative model [17]. As Dixon-Woods et al. found, inter-organisational communication and collaboration conveys strong normative pressure to engage with the programme and improve performance to match that of others [24].

The Australian Primary Care Collaborative (APCC) had been established in 2005; it involved over 4000 health professionals from over 2000 services across the country, with a principal goal of improving access and chronic disease care [43]. This initiative was running in parallel with the TORPEDO study and achieved some improvements in quality of care and clinical outcomes [43]. The TORPEDO team worked with the APCC group, using the APCC web platform for reporting peer-ranked data, and running joint workshops and webinars aimed at GP practices and ACCHSs. But the uptake of these efforts was variable and restricted to GP practices that were already experienced in the quality improvement collaborative approach [33]. Those practices aside, inter-organisational communication and networking was limited. Some of the TORPEDO team reflected on the tension between the RCT design (assumed to be a controlled experiment of a fixed intervention) and the more iterative approach encouraged in quality improvement:

there’s always the challenges of the RCT design, the side of you that you sort of test fixed ingredients or pills, and you don’t change things, adapt things over time. So I think if we had a different kind of design in evaluating this, it would have been more of that kind of cyclical adaptation over time, constantly reiterating, modifying our intervention, potentially taking it into different areas as we started to build a sort of community of practice, and I think all of those things are as important—ex post interview 2

### Adaptation over time

The TORPEDO study began in 2008, so this analysis allowed us to assess how *HealthTracker*, and the organisations seeking to support its use, had evolved and adapted over time. As noted in the previous section, a desire to keep the intervention fixed to meet the standards of the RCT design existed in tension with the need to make local adaptations to improve its embedding.

One challenge for practices was maintaining staff skills in the face of high turnover or flagging commitment. GPs who used *HealthTracker* only sporadically tended to forget the content of the training. Some practices found that it was necessary to retain “someone on the ground who is familiar with the tool inside out and with the IT infrastructure, who can coordinate with the developers” (ex post interview 1). Such support implies a recurrent cost, to be borne by Primary Health Networks or GP practices (or, within the context of the study, by the TORPEDO research group). Another factor that reduced sustainability of *HealthTracker* was the limited ability of the software vendor to respond technical difficulties by adapting the technology. It took around 2 years after the TORPEDO implementation study ended for them to release the next generation of the software (which GPs claimed still had “bugs”). This lack of agility had a negative impact on adoption. The TORPEDO team subsequently moved the development of *HealthTracker* in-house to a technology spinoff of their host research institute.

The limited interoperability of *HealthTracker* with other technical systems (see domain 2) was viewed by TORPEDO researchers as problematic in the context of more integrated clinical workflows within primary care and a national policy decision to increase interoperability between primary, secondary, and tertiary care. Some researchers felt that to make the technology more sustainable, it would need to develop the functionality to exchange information between systems rather than simply calculate and visualise risk. They considered that unless *HealthTracker* becomes fully integrated into the electronic record, it will inevitably have to compete with other third-party add-ons, as “ … one player in a very congested space, competing for that crowded real estate on the GP’s screen” (ex post interview 2). To address this challenge, researchers suggested expanding the number of conditions for which *HealthTracker* could be used: “if *HealthTracker* is … for just one condition, you might get a few people to use for a little while, but … if it could be developed for a whole range of interventions that might be sustainable … [for example if it had] multiple uses … like a Swiss Army Knife, … so that it looked the same and did similar things” (ex post interview 4). The counter-argument is that additional functionality would increase both technical and operational complexity and likely generate new problems elsewhere in the system.

An opportunity recently emerged to adjust financial incentives. In 2018, the entire Medicare Benefits System programme was undergoing a review (commenced in 2015), and an application for listing (not specific to *HealthTracker*) was made to create item numbers around performing risk assessment and management. A similar submission was recently also made to the Medical Services Advisory Committee, which advises the Australian government on which new medical services should receive public funding. As of April 2019, interim MBS items (to be reviewed over the next 2 years) have been introduced to allow GPs and non-specialist physicians to conduct a heart health check that lasts at least 20 min. This recent development has potential to shift the value proposition (see domain 4) for *HealthTracker* to make GPs’ use of the technology worthwhile.

Whist TORPEDO researchers were upbeat about the potential for increasing uptake of *HealthTracker* via such national-level levers, they acknowledged that “ … regulating clinical practice is difficult … ultimately, it’s always going to be optional, [as] the doctor can always say, I didn’t have time, I wasn’t interested, it didn’t seem like the right patient” (ex post interview 5). They also recognised that technologies generally do not have universal appeal: “Some people would [be interested], some people might not, it’s the same as almost any other thing, some practices have a spirometer and some don’t” (ex post interview 5)*.* And that if the choice on whether to adopt *HealthTracker* (or not) was left to individual GPs or GP practices, uptake would likely be slow, because GPs may only realise that the technology was helpful *after* they had started using it. Purchase by GP practices or Primary Health Networks in such a scenario would depend on price and competing third-party software.

Two changing features of the governance structure of Australian general practice may influence adoption of *HealthTracker* in the future. First, it is possible that Primary Health Networks will start to provide significant direct support to GP practices to implement quality improvement initiatives, though *HealthTracker* may or may not be prioritised in this move. Second, with the growth of corporatised GP practice chains, more practices will have key staff such as a practice manager, IT lead, and quality improvement lead. But as the TORPEDO team found in their experience with larger GP practices, buy-in from the CEO of such corporatised practices does not guarantee that front-line clinicians will use the tool.

Another potentially positive development on the horizon is policy support for new digital health initiatives. Whilst Australia has included digital health in strategic documents since around 2005, in 2017, the first National Digital Health Strategy was released. It named several relevant goals to be achieved by 2022: (1) digitally enabled care models to improve accessibility, quality, safety, and efficiency of care; (2) workforce confidently using digital health technologies; and (3) high-quality data with a common understood meaning that can be used with confidence [44]. However, there is still perceived to be a mismatch of investment decisions and activities needed at the organisational and adopter levels to address identified gaps in healthcare delivery and their links to improved population outcomes.

In sum, whilst there are some positive trends, there remains a high degree of uncertainty about how the fortunes of *HealthTracker*, both locally and nationally, will unfold in the future.

## Discussion

### Summary of empirical findings: what explains TORPEDO?

This ex post evaluation has identified a number of interacting explanations for *HealthTracker*’s varied and partial uptake. Before listing these, it is worth noting that whilst there were undoubtedly some weaknesses in the original TORPEDO studies, it is striking how many strengths were built into the design and implementation. The technology was developed through extensive co-design; the programme had strong leadership and clear goals; much effort was made to recruit practices working in areas where unmet need was high, and considerable support was provided to practices to set up the technology, train staff in its use, and support a collaborative approach to quality improvement. Despite these strengths, TORPEDO has, to date, had only a limited impact on patient outcomes. Below, we summarise our findings.

Cardiovascular risk is strongly influenced by social determinants and often coexists with comorbidities and entrenched lifestyle patterns; a technology designed to support rational decision-making based on epidemiological risk models may not appeal to many patients. *HealthTracker* had some significant software design flaws—e.g. it presupposed a level of technical infrastructure that some organisations did not possess. The value proposition for the technology’s vendor depended on widespread uptake across primary care providers, but because of the prevailing fee-for-service funding model in Australian general practice and lack of specific quality incentives for preventive care, there were costs associated with using *HealthTracker* for GP practices—for example, it required more time than was funded through Medicare standard consultations. Some GPs resisted using *HealthTracker* because its guideline-based recommendations conflicted with informally shared assumptions (mindlines) about best practice, and in such circumstances, design features (e.g. red lights) generated psychic costs [38].

Limited capacity to innovate (e.g. lack of infrastructure, skills, and support staff), mismatch of commitment between those signing the organisation up to the study and those who would be responsible for delivering on it, mismatch between implementation strategy (which was standardised) and widely varying capacity and governance structure of GP practices, and underestimating the work of implementation helped explain why some organisations were unable to fully integrate *HealthTracker* into business as usual. The wider institutional environment (professional, financial and regulatory), whilst not entirely adverse, was not sufficiently aligned and did not provide specific incentives, and inter-organisational networking occurred only to a limited extent. Most of these influences appear set to continue to pose challenges in the future, though recent realignments of financial incentives may positively influence the value proposition for GP practices.

### Summary of theoretical findings: how did the NASSS framework add value?

This study has also shown that the NASSS framework can be applied retrospectively to produce a new theorisation of a historical dataset which extends rather than replaces research and evaluations undertaken at the time. In particular, NASSS was built on the assumption that implementation of technologies in healthcare tends to follow the logic of complex systems [10, 20]. The seven NASSS domains are interdependent and interact in non-linear and unpredictable ways. Technologies designed to improve quality of care, even when programmed with the latest evidence-based guidelines, are not simple conduits for those guidelines, nor will their introduction *determine* particular behaviours or outcomes. Rather, technologies exert their influence (if at all) by becoming part of a dynamic network of people and other technologies which *generates* particular activities in particular contexts. Only when—and to the extent that—the “ensemble” of technologies-plus-people-in-wider-context comes together optimally will target patient groups *actually* receive better care and expect better outcomes [8, 45].

The “complex systems” analytic lens of the NASSS framework has also surfaced the tendency of technologies to “configure the user”. *HealthTracker* was designed by enthusiasts for evidence-based preventive care. Implicit in the software were assumptions—perhaps unintended and also unjustified—about the clinician (assumed to be a GP committed to following guidelines) and the patient (assumed to be a rational chooser with at least a moderate level of health literacy and numeracy). This systems lens also revealed that once a technology is installed in an organisation, there exists a greater or lesser potential to adapt and accommodate it. *HealthTracker*, for example, might have been better accommodated in Aboriginal Community Controlled Health Centres by creatively extending its use to community health workers who had ongoing relationships with patients and understood their cultural contexts (as opposed to restricting its use to temporary GPs who did not). This phenomenon (known as interpretive flexibility [45]) is critical to the successful embedding of technologies in organisational workflows and processes. The limited capacity to influence the institutional environment [46] and for organisational routines to adapt in the *HealthTracker* example suggests that the software and the organisations into which it was being introduced may have been too “brittle” to survive in the complex system of Australian general practice.

### Comparison with other literature

No previous studies have applied NASSS in an ex post analysis. The findings from this study resonate closely with our own and others’ application of NASSS in the empirical evaluation of health technology projects in the UK [20–23].

Dixon-Woods et al. applied a different theoretical lens to explain the success of the US Keystone Project [24] and the failed attempt to replicate this success in the UK [26]; they placed less emphasis on the technology and more on the various social practices and processes involved in the change effort. The six synergistic social influences that helped explain both the US success and UK failure of Keystone had some parallels in NASSS. For example, isomorphic pressures from other provider organisations would have been captured in domain 6 of the NASSS framework (extra-organisational influences). These pressures were weak in the TORPEDO study because most practices were not familiar with, or participating in, collaborative quality improvement approaches, and because of the Royal Australasian College of GPs’ ambivalence towards the technology.

Dixon-Woods et al.’s emphasis on the social and behavioural aspects of the intended change is captured in domain 2 of the NASSS framework (focused on staff concerns and professional codes of practice) and also domain 5 (specifically, “work needed to plan, implement, and monitor change”). The TORPEDO study had included little in the way of behavioural intervention because the research team were cautious about providing too much support since the resource implications would then make the intervention unscalable. Another finding from Dixon-Wood et al.’s analysis of Keystone was the importance of nurturing a culture of commitment to quality improvement. This was captured in domain 5 of the NASSS framework as part of the work to support change; in TORPEDO, maintaining such a culture was something of an uphill struggle in the absence of specific financial incentives.

Finally, Dixon-Woods’ finding that harnessing performance data as a “disciplinary force” and the use of “hard-edged formal accountability” are reflected in domain 6 of the NASSS framework as external (regulatory) influences on the system. In TORPEDO, a major motivator for many GPs was the peer-ranked performance portal described above, but the accountability was not “hard-edged”, since TORPEDO was run as a research study on collegiate lines, not as a policy must-do. The comparison with the Keystone Project highlights the tricky trade-offs that must be made in RCTs of complex interventions between undertaking a theoretically “robust” RCT and taking steps to maximise real-world success.

### Strengths and limitations of the NASSS framework for ex post evaluation

The NASSS framework has proved useful in understanding how and why a technology-enabled quality improvement intervention generated mixed outcomes. Earlier evaluations of the programme, including a randomised controlled trial [30], process evaluation [29], qualitative explorations of patients’ and clinicians’ experiences [27, 31], real-world implementation study of sustainability post-trial [32], organisational-level theorisation using normalisation process theory [33], and an economic evaluation (Patel et al., submitted), all contributed valuable insights. Re-theorising these various findings through the NASSS framework added insights at the overall health system level, illustrating the interplay between the various contributory factors at different levels and the specific local environments in which they played out.

The limitations of using the NASSS framework as an ex post analytic tool are similar to using any retrospective approach to undertake research. Apart from the narratives of long-standing research staff (which may be affected by recall bias), the dataset already exists and cannot be extended with new, real-time data. In a large, longitudinal study such as TORPEDO, material that could have enhanced a system-wider analysis might have been inadvertently discarded at the time by researchers operating a more deterministic paradigm.

## Conclusion

The NASSS framework, originally developed to explain the fortunes of health technology projects in real time, can be applied retrospectively to generate a rich, contextualised narrative of a technology-supported change effort and the numerous interacting influences on its successes, failures, and unexpected events. A NASSS-informed ex post analysis, drawing on the principles of complex systems, can supplement earlier contemporaneous evaluations to uncover emergent interactions and interdependencies that were not fully knowable or predictable at the time.

Whilst it is widely recognised that technology implementation in healthcare requires a judicious mix of “top-down” [47], “bottom-up” [48], and “middle-out” approaches [49], the literature still lacks rich exemplar case studies of how such approaches may dovetail (or not) in practice. Whilst not the only way to approach complexity in technology implementation, NASSS can be used to generate multi-level accounts that incorporate the target health condition(s), the technology, the adopter system (patients, providers, managers), the organisational elements, and the broader system enablers (policy, financing, etc.). Explaining in rich detail why past programmes succeeded or failed potentially allows us to learn from history and improve the design of future programmes.

We are currently extending the NASSS framework alongside a complexity assessment tool (CAT) for use as an ex ante tool for planning, managing, and evaluating complex technology projects in health and social care. Further details of the NASSS-CAT tool are available from the corresponding author.

## Data Availability

The TORPEDO dataset is held and managed by the George Institute of Global Health, University of New South Wales, Sydney, Australia. All inquiries should be addressed to Professor Anushka Patel who was the Chief Investigator of the study.
